# d-Tryptophan enhances the reproductive organ-specific expression of the amino acid transporter homolog *Dr-SLC38A9* involved in the sexual induction of planarian *Dugesia ryukyuensis*

**DOI:** 10.1186/s40851-021-00173-z

**Published:** 2021-03-20

**Authors:** Takanobu Maezawa, Masaki Ishikawa, Kiyono Sekii, Go Nagamatsu, Ryohei Furukawa, Kazuya Kobayashi

**Affiliations:** 1 Advanced Science Course, Department of Integrated Science and Technology, National Institute of Technology, Tsuyama College, 624-1 Numa, Tsuyama, Okayama 708-8509 Japan; 2 Faculty of Agriculture and Life Science, Hirosaki University, 3 Bunkyo-cho, Hirosaki, Aomori 036-8561 Japan; 3 Department of Stem Cell Biology and Medicine, Graduate School of Medical Science, Kyushu University, 3-1-1 Maidashi, Higashi-ku, Fukuoka, 812-8582 Japan; 4 Department of Biology, Research and Education Center for Natural Sciences, Keio University, 4-1-1 Hiyoshi, Kohoku-ku, Yokohama, Kanagawa 223-8521 Japan

**Keywords:** Planarian, Sexual induction, Germ cell, Amino acid transporter, Tryptophan

## Abstract

**Background:**

Many animals switch between asexual and sexual reproduction in nature. We previously established a system for the sexual induction of planarian *Dugesia ryukyuensis* by feeding asexual planarians with minced sexual planarians. We identified dl-tryptophan (Trp) as one of the sex-inducing substances. dl-Trp can induce ovarian development, the first and essential step of sexual induction. d-Trp must act as a principal bioactive compound in terms of ovarian development, because the ovary-inducing activity of d-Trp was 500 times more potent than that of l-Trp. However, how Trp controls sexual induction is still unknown.

**Results:**

In this study, qRT-PCR analyses suggested that the putative amino acid transporter gene *Dr-SLC38A9* is highly expressed in sexual worms, especially in the yolk glands. In situ hybridization analyses showed that *Dr-SLC38A9* is expressed in the ovarian primordia of asexual worms and in the mature ovaries, testes, and yolk glands of sexual worms. In addition, *Dr-SLC38A9* RNA interference during sexual induction resulted in the suppression of the development of reproductive organs. These results suggest that *Dr-SLC38A9* is involved in the development of these organs. Moreover, we demonstrated that the reproductive organ-specific expression of *Dr-SLC38A9* is enhanced by the addition of d-Trp.

**Conclusion:**

We propose that d-Trp activates the expression of *Dr-SLC38A9* to promote sexual induction in the planarian *D. ryukyuensis*.

**Supplementary Information:**

The online version contains supplementary material available at 10.1186/s40851-021-00173-z.

## Background

Many animals switch between asexual and sexual reproduction, depending on environmental conditions and/or the phase of their life cycle [1–4]. The switching of reproductive modes might be a way to take advantage of both forms of reproduction. However, the underlying mechanisms are poorly understood [1].

Certain freshwater planarians reproduce both asexually and sexually. Asexual planarians reproduce by dividing their body into two parts: each part regenerates the lost half. This regenerative ability is afforded by pluripotent stem cells known as neoblasts [5, 6]. Sexual planarians possess hermaphroditic reproductive organs and copulate with other sexual planarians. Reproductive organs are differentiated from neoblasts postembryonically, in the juvenile stage [7]. Some asexual planarians may develop hermaphroditic reproductive organs from neoblasts in the adult stage and undergo sexual reproduction, depending on environmental conditions [8–13]. Reproductive mode switching rarely occurs under controlled laboratory conditions. Therefore, methods to induce sexual development in asexual worms have contributed to our understanding of reproductive switching [14–17]. We previously established a biological assay system to sexually switch an asexual clonal population of *Dugesia ryukyuensis* (OH strain) by feeding with the minced bodies of sexually mature worms of the species *Bdellocephala brunnea* [1]. The occurrence of the switch indicates that sexually mature worms contain one or more chemicals that stimulate sexual induction in OH worms, and we defined these chemical(s) to be sex-inducing substance(s). During sexual induction, within approximately 1 month, asexual worms develop a pair of ovaries, yolk glands, testes, copulatory apparatus, and a genital pore, in that order (Fig. S1) [18, 19]. This sex-induction process is divided into five stages (Fig. S1) [18, 19]. Sexually mature worms successfully copulate with each other and eventually lay cocoons (ectolecithal eggs) containing several fertilized eggs and numerous yolk gland cells. Yolk gland cells are produced in the yolk gland, which is a reproductive organ unique to planarians [20, 21].

In a previous study, we found that the feeding of fresh cocoons containing numerous yolk gland cells to asexual worms caused their full sexual induction [22]. This result suggests that yolk gland cells might contain sex-inducing substance(s) that trigger full sexual induction. We carried out bioassay-guided fractionation to identify the sex-inducing substance(s) contained in *B. brunnea* and identified tryptophan (Trp) as a sex-inducing substance based on electrospray ionization-mass spectrometry (ESI-MS) and nuclear magnetic resonance (NMR) analyses [23]. Furthermore, we found that *B. brunnea* contained 0.5% d-Trp relative to l-Trp (w/w) by reverse-phase high-performance liquid chromatography (HPLC). Through feeding dl-Trp, asexual worms were induced to stage 1–2 worms presenting immature ovaries, which suggests that dl-Trp is involved in ovarian development, the first step of sexual induction. Interestingly, d-Trp must act as a principal bioactive compound in terms of ovarian development, because the ovary-inducing activity of d-Trp was 500 times more potent than that of l-Trp. However, how Trp controls sexual induction is still unknown. We found that Trp is enriched in the yolk glands of stage 5 (sexual) worms of *D. ryukyuensis* [23]. Trp is an essential amino acid and cannot be produced in animal bodies. Considering the enrichment of Trp in yolk glands and the ovary-inducing activity of Trp, we hypothesized that ovaries and yolk glands express a specific amino acid transporter. Therefore, in the present study, we isolated a candidate amino acid transporter gene and analyzed its expression and function during sexual induction.

## Methods

### Animals

An exclusively asexual strain of the planarian *D. ryukyuensis* (OH strain) [24, 25] was established by Dr. S. Ishida at Hirosaki University (Hirosaki, Japan). The OH strain was cultured at 20 °C in dechlorinated tap water. Asexual worms were fed chicken liver once per week. Sexual *D. ryukyuensis* worms were induced from asexual worms by feeding them minced *B. brunnea* worms [26]. In the sexual induction assay, five individuals of approximately 5 mm in body length were placed in a 90 mm plastic dish and fed daily with 10 mg (wet weight) of minced *B. brunnea* worms for a month [1].

### Cloning of *D. ryukyuensis SLC38A9* cDNA

Total RNA was extracted from sexual *D. ryukyuensis* worms using the Sepasol RNA I Super Extraction Kit (Nacalai Tesque, Kyoto, Japan). First-strand cDNA was prepared from total RNA using random primers and reverse transcriptase (Toyobo, Osaka, Japan). The cDNA of *D. ryukyuensis SLC38A9* (*Dr-SLC38A9*) was amplified by PCR using first-strand cDNA as a template and the primers 5′- CCGGAACGCAGAGTAGTTTC − 3′ (forward) and 5′- GCCGAGAGCATTAAATTTGG − 3′ (reverse). This primer set was designed based on the sequence of *TR38642|c0_g1_i2* from RNA-seq of *D. ryukyuensis* [27]. The PCR protocol was as follows: 94 °C for 2 min, followed by 30 cycles of 94 °C for 30 s, 55 °C for 30 s, and 68 °C for 1 min. Full-length sequences of the genes were obtained using 3′ and 5′- rapid amplification of cDNA ends (RACE) using the SMART™ RACE cDNA Amplification kit (Clontech, Mountain View, CA, USA) and the following primer set: *Dr-SLC38A9*_5RACE, GTTCGACCGGGCTCCAGAGGTAAAT; and *Dr-SLC38A9*_3RACE, 5′-TGATCACTTTGCCCGCATTGGTG-3′. The nucleotide sequence of *Dr-SLC38A9* reported in this paper has been deposited in the DDBJ nucleotide sequence database (accession number LC225747; https://www.ddbj.nig.ac.jp/index-e.html). Homology searches for Dr-SLC38A9 were performed using the NCBI BLASTp program (https://blast.ncbi.nlm.nih.gov/Blast.cgi). We obtained sequences of SLC38A9 homologs in humans, mice, and zebrafish and of SLC38 family members in the planarian *Schmidtea mediterranea* from the NCBI database (https://www.ncbi.nlm.nih.gov). Phylogenetic trees of human and planarian *S. mediterranea* SLC38 proteins [28] and Dr-SLC38A9 of planarian *D. ryukyuensis* were constructed using the neighbor-joining method [29] in ClustalW (https://www.genome.jp/tools-bin/clustalw). Protein sequences were aligned using ClustalW.

### Quantitative reverse-transcription PCR

Quantitative reverse-transcription PCR (qRT-PCR) was performed using the 7300 Real-Time PCR System (Applied Biosystems, Foster City, CA, USA). Each reaction mixture (25 μL) contained 12.5 μL of Power SYBR Green PCR Master mix (Applied Biosystems), 0.4 μM gene-specific primers, and 0.5 μL of cDNA template. Planarian cDNA was prepared as described above. The PCR protocol was as follows: 50 °C for 2 and 95 °C for 10, followed by 40 cycles of 95 °C for 15 s and 60 °C for 1 min. Measurements were normalized to the expression levels of glyceraldehyde 3-phosphate dehydrogenase homolog (*GAPDH*) [30] or elongation factor 1 alpha homolog (*Dr-ef1a*) [31]. The primers used in the current study are shown in Supplementary Table S1. The raw qRT-PCR analysis data are shown in Supplementary Data Sheet S1.

### Statistical analysis

**Fig. 1:**
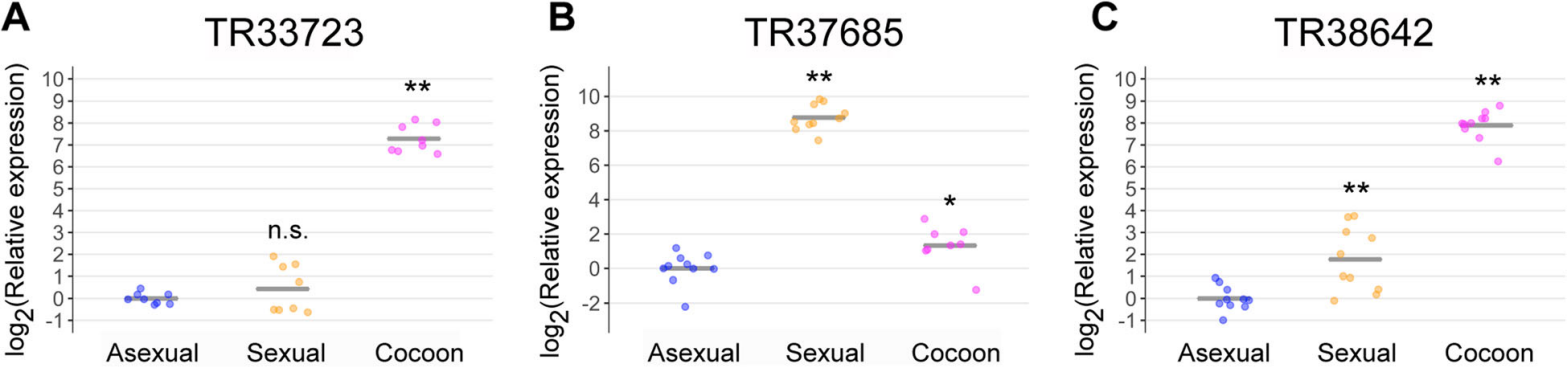
Comparison of TR33723, TR37685, and TR38642 mRNA levels in asexual worms, sexual worms, and cocoons. qRT-PCR data are shown relative to the expression level (normalized to the *Dr-ef1a* mRNA level) in the asexual worm; log_2_ (relative expression) on the vertical axis indicates −∆∆Ct. Each circle indicates an individual asexual worm, sexual worm, or cocoon sample. The bars in the plots indicate the average − ∆∆Ct values. Asterisks indicate significant differences compared with asexual worms (Tukey’s HSD test: **P < 0.01; ***P < 0.001)

The between-sample differences in obtained threshold cycle (Ct) values were calculated using the ∆∆Ct method. Briefly, ∆Ct [where ∆Ct = Ct (target gene) – Ct (internal control)] was calculated for each sample, and then ∆∆Ct [where ∆∆Ct = ∆Ct (sample) − the average of ∆Ct (calibrator)] was calculated. Calibrators were asexual worms (Fig. 1), EGFP knockdown worms (Fig. 4) and liver-fed worms (Fig. 5). Statistical tests were performed on the ∆∆Ct values. Relative expression was calculated as 2^−∆∆Ct^. Statistical tests were performed using R v3.2.2 [32]. The Shapiro-Wilk test was used to validate the normal distributions of the obtained data, and the F-test or Bartlett’s test was used to validate the equality of variances. Student’s t-test was used to compare gene expression levels between two groups. In some cases, Welch’s t-test was used because of unequal variances between the samples. To compare gene expression levels between three groups, one-way ANOVA with post hoc comparison using Tukey’s honestly significant difference (HSD) test was conducted.

### In situ hybridization and histological analysis

In situ hybridization of whole-mount worms and sections was performed according to our previously published methods [30, 33]. Sagittal 4-μm thick sections were used for in situ hybridization and for hematoxylin and eosin (HE) staining. For in situ hybridization, digoxigenin (DIG)-labeled antisense RNA probes were synthesized using the MEGAscript Kit (Ambion, Austin, TX, USA) from a 695-bp fragment of the open reading frame (ORF, 329–1023) of *Dr-SLC38A9*. A probe for *Dr-nanos* was synthesized as previously described [34]. Both probes were hydrolyzed with an equal amount of carbonated buffer (80 mM NaHCO_3_, 120 mM Na_2_CO_3_, 10 mM DTT) at 60 °C for 60 min, neutralized and ethanol precipitated. DIG was detected using an alkaline phosphatase (AP)-conjugated anti-DIG antibody (Roche, Mannheim, Germany) and stained blue with NBT/BCIP solution [17 μg/mL nitro-blue tetrazolium chloride (NBT) and 8.8 μg/mL 5-bromo-4-chloro-3-indolyl phosphate, toluidine-salt (BCIP)]. The slides were dipped in 1 μg/mL Hoechst No. 33342 (Sigma, St. Louis, MO, USA) to counterstain the nuclei.

### RNA interference analysis

Double-stranded RNA (dsRNA) was synthesized in vitro using the MEGAscript High Yield Transcription kit. The *Dr-SLC38A9* dsRNAs were a 529-bp fragment (399–927) and 521-bp fragment (1053–1573) of the ORF. The *enhanced green fluorescent protein (EGFP)* dsRNA was the full-length ORF (720 bp). These dsRNAs were purified using phenol/chloroform and then annealed. To prepare the test foods used for the knockdown experiments under sex-inducing conditions, dsRNAs were added to minced *B. brunnea* and stored at − 80 °C until use. The worms were fed twice at 2-day intervals with chicken liver supplemented with 200 ng of *EGFP* or *Dr-SLC38A9* dsRNA. On 1 day after the second feeding, the posterior and anterior regions of the worms were amputated, and the trunk fragments were incubated without food for 7 days. The partially regenerated worms were then fed chicken liver supplemented with 200 ng of *EGFP* or *Dr-SLC38A9* dsRNA twice at 2-day intervals to allow the worms to fully regenerate. The resultant asexual worms were then fed minced *B. brunnea* supplemented with 200 ng of the dsRNAs of the indicated genes daily for 6 weeks.

### Preparation of test foods containing sex-inducing substances for the induction of *Dr-SLC38A9* expression

The M0 + M10 fraction, which has sex-inducing activity, was prepared from extracts of *B. brunnea* worms (1 g wet weight) as previously described [19]. We homogenized *B. brunnea* worms in phosphate-buffered saline (PBS) and obtained a cytosolic fraction after ultracentrifugation. The cytosolic fraction was desalted and eluted stepwise by changing the methanol concentration of the eluent (0, 10, and 100% (v/v)) on a commercial octadecylsilane (ODS) column, Sep-Pak® Light tC_18_ Cartridge (Waters, Milford, MA). Strong sex-inducing activity was recovered in the fractions eluted with water and 10% methanol (M0 fraction and M10 fraction) [19]. The M0 + M10 fraction was dried, mixed with 100 μL of chicken liver homogenate, and freeze-dried. The food was divided into three portions and used for the experiments. Fifteen sexual worms were fed a portion of food daily over 3 days. After 3 days of treatment, sexual worms were subjected to whole-mount in situ hybridization or qRT-PCR. The l-Trp and d-Trp contents in the M0 + M10 fraction from *B. brunnea* (4 g wet weight) were estimated to be approximately 2 mg and 10 μg, respectively [23]. Therefore, in this experiment, commercial l-Trp (500 μg; Nacalai Tesque, Kyoto, Japan) and/or d-Trp (2.5 μg; Nacalai Tesque, Kyoto, Japan) were mixed with a 100-μL aliquot of chicken liver homogenate, and the respective mixtures were freeze-dried. The food was divided into three portions and used for the experiments. Fifteen sexual worms were fed together with a daily portion of food for over 3 days. After 3 days of treatment, sexual worms were examined by qRT-PCR.

## Results

### Isolation of the putative amino acid transporter gene *Dr-SLC38A9* in *D. ryukyuensis*

**Fig. 2:**
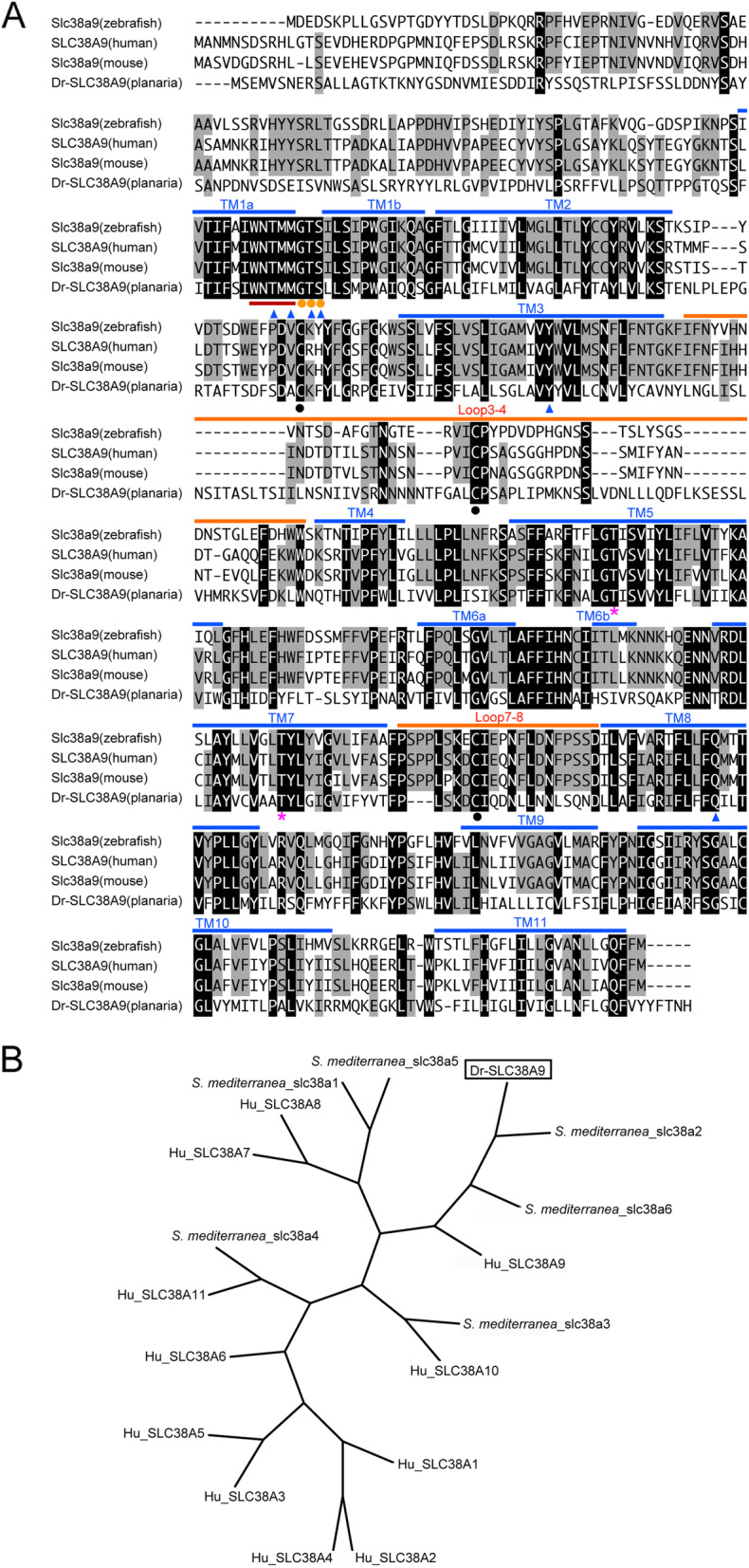
Alignment of SLC38A9 protein homologs from four animal species and phylogenetic tree of SLC38 proteins. **a** Multiple sequence alignment of animal SLC38A9 homologs in zebrafish (Slc38a9: NP_001073468), humans (SLC38A9: AAI01363), mice (Slc38a9: GenBank accession number NP_848861), and planarians (Dr-SLC38A9: LC225747) showed several conserved amino acid residues. Black boxes indicate residues that were identical in all aligned proteins. Gray backgrounds indicate residues that were identical in three aligned proteins. Blue lines, transmembrane regions; brown lines, loops bound to Fab; red underline, WNTMM anchor motif; orange circles, GTS conserved motif; magenta asterisks, residues bonded with TM1a; blue triangles, residues involved in Arg binding; black circles, disulfide bonds between loops 3–4 and loops 7–8. **b** Phylogenetic tree of human SLC38 proteins (SLC38A1: GenBank accession number NP_001070952, SLC38A2: NP_061849, SLC38A3: NP_006832, SLC38A4: NP_001137296, SLC38A5: NP_277053, SLC38A6: NP_722518, SLC38A7: NP_001356537, SLC38A8: NP_001073911, SLC38A9: NP_001336311, SLC38A10: NP_001033073, SLC38A11: NP_001186077), the planarian *Schmidtea mediterranea* (*S. mediterranea*_slc38a-1: AKN21660, *S. mediterranea*_slc38a-2: AKN21661, *S. mediterranea*_slc38a-3: AKN21662, *S. mediterranea*_slc38a-4: AKN21663, *S. mediterranea*_slc38a-5: AKN21664, *S. mediterranea*_slc38a-6: AKN21665) [28], and Dr-SLC38A9 of the planarian *Dugesia ryukyuensis,* constructed using the neighbor-joining method [29]

We searched for amino acid transporter genes enriched in sexual planarians using the RNA-seq data of *D. ryukyuensis* [27]. We focused on the DEG (differentially expressed gene) data between asexual and sexual planarians and identified three annotated amino acid transporter genes (*TR33723|c0_g1_i1*, vesicular inhibitory amino acid transporter; *TR37685|c0_g2_i3,* excitatory amino acid transporter 2; and *TR38642|c0_g1_i2,* sodium-coupled neutral amino acid transporter 9 isoform × 1). Next, we carried out q-PCR analysis using primers specific to these genes on cDNAs derived from asexual worms, sexual worms, and cocoons (Fig. 1a, b, c). One-way between-subjects ANOVA was conducted to compare the effect of sexual condition on *TR33723|c0_g1_i1*, *TR37685|c0_g2_i3*, and *TR38642|c0_g1_i2* gene expression. There was a significant effect of sexual condition on gene expression with *P* < 0.001 for the three conditions, with [F(2, 21) = 243.9, *P* = 2.92e-15], [F(2, 25) = 236.6, *P* < 2e-15], and [F(2, 27) = 173.3, *P* = 3.96e-16], respectively. Next, to compare the gene expression levels among the three groups, Tukey’s honestly significant difference (HSD) test was used. The expression levels in cocoons indicates those in yolk glands because cocoons are mostly filled with yolk gland cells. Considering that tryptophan is enriched in yolk glands [23], we searched for genes that were more highly expressed in cocoons than in asexual and sexual worms, such as *Dryg*, which is a yolk gland marker gene (Fig. 3S of [35]). Although the relative expression level of *TR37685|c0_g2_i3* was significantly higher in both sexual worms (437-fold) and cocoons (2.51-fold) than in asexual worms (Fig. 1b), this expression pattern was not identical to that of *Dryg*. Thus, *TR37685|c0_g2_i3* seems to be expressed in sexual organs other than the yolk glands. The expression of this gene in the cocoons could be attributed to the presence of fertilized eggs. In contrast, as the expression patterns of *TR33723|c0_g1_i1* and *TR38642|c0_g1_i2* are very similar to that of *Dryg*, it is expected that these genes will be expressed in yolk glands. Additionally, we sought target genes that are expressed in at least ovaries and yolk glands. The relative expression level of *TR33723|c0_g1_i1* in cocoons (156-fold) was significantly higher than that in asexual worms. However, the expression in sexual worms was not significantly different from that of asexual worms (1.36-fold) (Fig. 1a). The relative expression level of *TR38642|c0_g1_i2* was significantly higher in both sexual worms (3.41-fold) and cocoons (238-fold) than in asexual worms (Fig. 1c). Given the potentially higher expression of this gene in yolk glands and ovary-expressed genes, in this study, we decided to focus on *TR38642|c0_g1_i2*. To confirm the sequence of *TR38642|c0_g1_i2*, we cloned full-length cDNA from *TR38642|c0_g1_i2* mRNA using reverse transcription PCR (RT-PCR) and RACE analyses. The cloned cDNA contains an open reading frame encoding a 585 amino acid (aa) polypeptide that has high homology with human SLC38A9 (73% identity) [36, 37] (Fig. S2). Therefore, we named this gene *Dugesia ryukyuensis SLC38A9* (*Dr-SLC38A9*). We found that the deduced amino acid sequence of the cloned cDNA has a longer N-terminal region than that of *TR38642|c0_g1_i2* from RNA-seq data. Fig. S2 shows the cloned nucleotide sequence and the deduced amino acid sequence of *Dr-SLC38A9*. Multiple sequence alignment of SLC38A9 homologs from different species (zebrafish, humans, and mice) showed many conserved amino acid residues (Fig. 2a). Previously, broadly conserved domains among SLC38A9 homologs were characterized by structural analysis of the zebrafish Slc38a9 protein [38]. Dr-SLC38A9 has a sequence similar to that of the 11-amino acid transporter and transmembrane domains that are conserved among SLC38A9 homologs (Fig. 2a) [36–38]. Dr-SLC38A9 also has a WNTMM anchor motif, which is important for the transport of arginine, and a GTS conserved motif (Fig. 2a). Next, we constructed a phylogenetic tree by comparing the sequence of Dr-SLC38A9 with representative SLC38 family members in humans and the planarian *Schmidtea mediterranea* (Fig. 2b). This phylogenetic tree shows that among other human SLC38 family members, the sequence of SLC38A9 is closest to that of Dr-SLC38A9. In *S. mediterranea,* six SLC38 family member genes have been reported [28]. In comparison with the *S. mediterranea* genes, the sequence of Dr-SLC38A9 was closest to that of *S. mediterranea*_slc38a2 (Fig. 2b). We also confirmed that SLC38A9 is the human protein with the greatest homology to *S mediterranea*_slc38a2 (38% identity).

### Expression analyses of *Dr-SLC38A9*

**Fig. 3:**
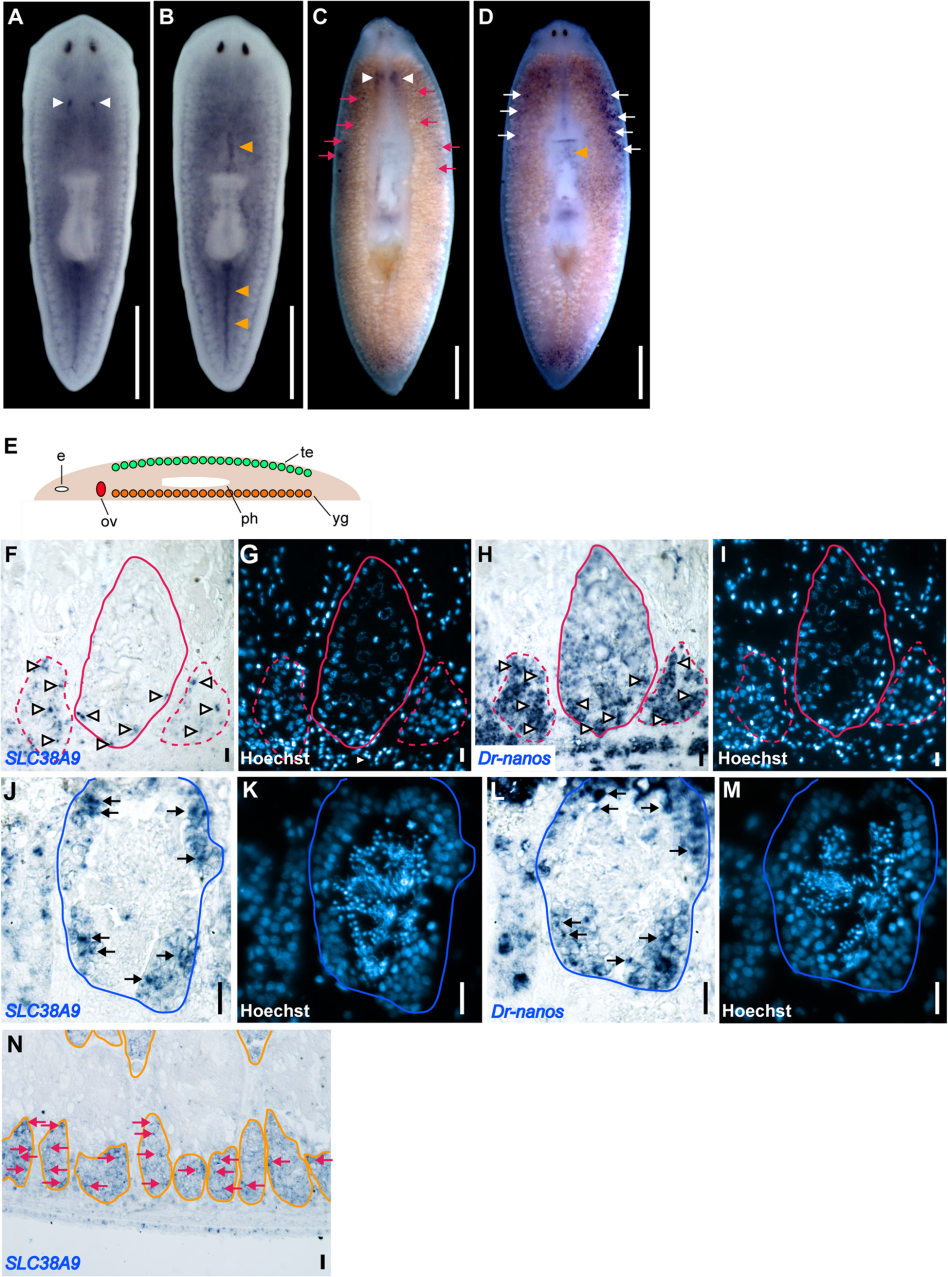
Dr-SLC38A9 mRNA expression in asexual and sexual worms. **a**-**d** Asexual (**a**, **b**) and sexual worms (**c**, **d**) were hybridized with an antisense probe to *Dr-SLC38A9* mRNA. *Dr-SLC38A9* transcripts are stained blue. Ventral views (**a**, **c**) and dorsal views (**b**, **d**) are shown with the anterior part of the worm at the top. White arrowheads indicate signals in the ovarian primordia (**a**) and mature ovaries (**c**). Orange arrowheads indicate signals in the parenchyma of the dorsal midline (**b**). Magenta arrows indicate signals in the yolk glands (**c**). White arrows indicate signals in the mature testes (**d**). Scale bars, **a**-**d** 1 mm. **e** Schematic of a sagittal section of a sexual planarian: e, eye; ov, ovary; ph, pharynx; te, testis; yg, yolk gland. **f**-**n** In situ hybridization of adjacent sections using an antisense probe to *Dr-SLC38A9* (**f**, **j**, **n**) or *Dr-nanos* (**h**, **l**) mRNA. **g**, **i**, **k**, **m** Nuclei in blue. The anterior part of the worm is to the left, and the dorsal part is at the top. **f** *Dr-SLC38A9* transcripts were detected at the periphery of the main ovary (magenta circle) and in satellite cells (magenta dotted circles) next to the main ovary. **h** *Dr-nanos* transcripts were detected at high levels in satellite cells (magenta dotted circle) next to the main ovary (magenta circle) and at low levels in the main ovary. **j**, **l** *Dr-SLC38A9* and *Dr-nanos* transcripts were detected in spermatogonia at the periphery of the testes (blue circle). (N) *Dr-SLC38A9* transcripts were widely detected throughout the yolk glands (orange circles). White arrowheads indicate signals of *Dr-SLC38A9* (**f**) and *Dr-nanos* (**h**) in mature ovaries. Black arrows indicate signals of *Dr-SLC38A9* (**j**) and *Dr-nanos* (**l**) in the mature testes. Magenta arrows indicate signals in the yolk glands (**n**). **h**, **l** We also detected nonspecific *Dr-nanos* signals in the epidermis*.* Scale bars, **f**-**n** 20 μm

To examine the expression pattern of *Dr-SLC38A9* in planarians, we performed whole-mount in situ hybridization (WISH) analyses on asexual and sexual worms. In asexual worms, *Dr-SLC38A9* transcripts were highly expressed in the ovarian primordia on the ventral side of the worm (Fig. 3a, white arrowheads) and in the dorsal midline at the anterior and posterior regions of the pharynx (Fig. 3b, orange arrowheads), while their expression was detected weakly throughout the parenchyma (Fig. 3a, b). In sexual worms, *Dr-SLC38A9* transcripts were also detected in mature ovaries at the ventral side (Fig. 3c, white arrowheads) and in the dorsal midline at the prepharyngeal region (Fig. 3d, orange arrowhead). In addition, *Dr-SLC38A9* transcripts were apparent in mature testes on the dorsal side (Fig. 3d, white arrows) and yolk glands on the ventral side (Fig. 3c, magenta arrows). To confirm the histological distribution of *Dr-SLC38A9* in testes and yolk glands, we carried out in situ hybridization analyses in sagittal sections of sexual worms. In the ovaries, *Dr-SLC38A9* transcripts were detected in early-stage oogonia or female germline stem cells at the periphery of the main ovaries (Fig. 3f, arrowheads in circles and Fig. 3g) and satellite cells next to the main ovaries (Fig. 3f, arrowheads in dotted circles and Fig. 3g), where the germline marker *Dr-nanos* was strongly expressed (Fig. 3h, arrowheads and Fig. 3i) [34]. In the testes, *Dr-SLC38A9* transcripts were detected in the spermatogonia at the periphery of the testes (Fig. 3j, arrows in a circle and Fig. 3k), where the germline marker *Dr-nanos* was also expressed (Fig. 3l, arrows in a circle and Fig. 3m). These results suggest that *Dr-SLC38A9* is highly expressed in early-stage germline cells during gametogenesis. In contrast, *Dr-SLC38A9* was widely expressed throughout the yolk glands (Fig. 3n, arrows in circles) in sexual worms.

### Function of *Dr-SLC38A9* in reproductive development during sexual induction

**Fig. 4:**
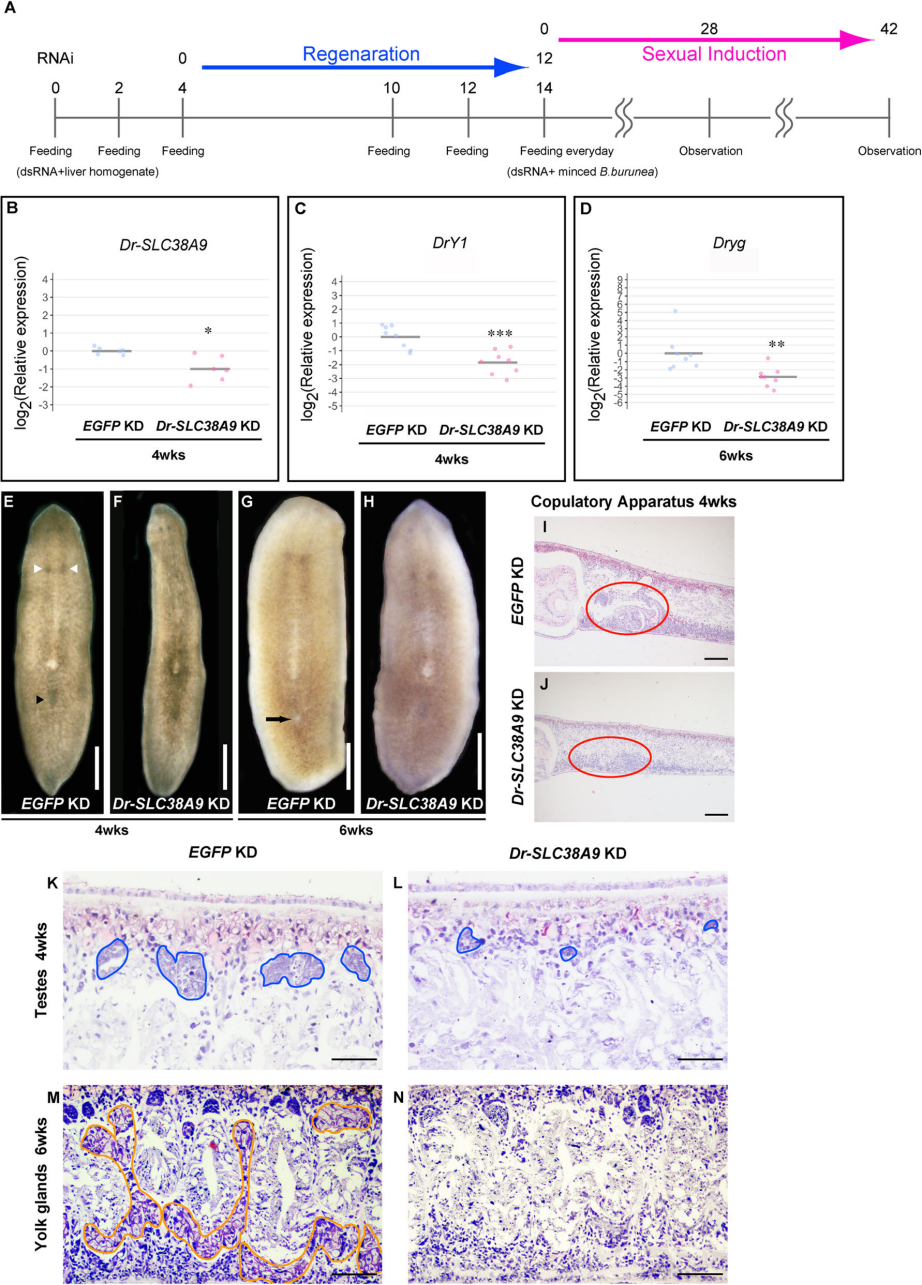
Dr-SLC38A9 knockdown phenotypes under sex-inducing conditions. **a** Scheme of an experimental schedule of RNA interference (RNAi) under sexual induction. **b**-**d** qRT-PCR data are shown relative to the expression level (normalized to *Dr-ef1a* mRNA level) in the *EGFP* KD worm; log_2_ (relative expression) on the vertical axis indicates −∆∆Ct. Each circle indicates an individual *EGFP* KD worm or *Dr-SLC38A9 KD* worm sample. The bars in the plots indicate the averages of −∆∆Ct. **b** *Dr-SLC38A9* transcript abundance after 4 weeks of treatment. Six replicates were performed. Significance was calculated using Welch’s *t*-test (*P < 0.05). **c** *DrY1* transcript abundance after 4 weeks of treatment. Significance was calculated using Student’s *t*-test (***P < 0.001). **d** *Dryg* transcript abundance after 6 weeks of treatment. Significance was calculated using Student’s *t*-test (**P < 0.01). **e**, **f** Development of ovaries (white arrowheads) and copulatory apparatus (black arrowhead). A ventral view is shown, with the anterior of the worm at the top. **g**, **h** Development of the genital pore (black arrow). A ventral view is shown, with the anterior of the worm at the top. Scale bar, **e**-**h** 1 mm. **i**-**l** Transverse sections from asexual worms fed daily with minced *B. brunnea* worms supplemented with *EGFP* (control; **i**, **k**, **m**) or *Dr-SLC38A9* (**j**, **l**, **n**) dsRNA for 6 weeks. The anterior part of the worm is to the left, and the dorsal part is at the top. **i**, **j** HE staining of the copulatory apparatus (red circles) after 4 weeks of treatment. **k**, **l** HE staining of the testes (blue circles) after 4 weeks of treatment. **m**, **n** HE staining of the yolk glands (orange circles) after 6 weeks of treatment. Yolk glands did not develop in the *Dr-SLC38A9*-knockdown worms. Scale bars, **i**, **j** 200 μm, **k**, **l** 50 μm, **m**, **n** 100 μm

To investigate the role of Dr-SLC38A9 in sexual induction, we performed *Dr-SLC38A9* knockdown experiments using RNA interference (RNAi) (summarized in Fig. 4a). RNAi was initiated from asexual worms. The animals were amputated once and regenerated after feeding with liver homogenate supplemented with *Dr-SLC38A9* or *EGFP* (control) dsRNA twice. *Dr-SLC38A9* knockdown worms were able to regenerate normally. Knockdown with sexual induction was then performed using minced bodies of sexually mature *B. brunnea* supplemented with *Dr-SLC38A9* or *EGFP* dsRNA. Expression analyses and histological analyses were carried out after 4 weeks and 6 weeks of this treatment, time points corresponding to stages 3 and 4 in terms of the development of the copulatory apparatus and genital pore, respectively. After 4 weeks of this treatment under sexual induction, we confirmed the reduced levels of *Dr-SLC38A9* mRNA by qRT-PCR (Fig. 4b, [mean (*EGFP* KD) = 1.00, mean (*Dr-SLC38A9* KD) = 0.549, t(5.7) = 3.31, *P* = 0.017]). We examined the development of ovaries and the copulatory apparatus in *Dr-SLC38A9-*knockdown worms externally using a stereomicroscope. After 4 weeks of treatment, the percentage of *Dr-SLC38A9-*knockdown worms that had developed ovaries and copulatory apparatuses was significantly lower than that of *EGFP*-knockdown (control) worms (Table 1, Fig. 4e, f). Moreover, histological examination revealed that the copulatory apparatus of *Dr-SLC38A9* knockdown worms was immature (Fig. 4j) compared with that of control worms (Fig. 4i). These results suggest that *Dr-SLC38A9* is involved in the development of ovaries and the copulatory apparatus during sexual induction. Next, we investigated the development of reproductive organs that could not be observed externally by qRT-PCR of *DrY1*, testis marker gene (previously reported as DrC_00456 [31, 34]) and *Dryg*, yolk gland marker gene [35], and through histological examination. *Dr-SLC38A9-*knockdown worms contained significantly lower levels of the testis marker *DrY1* than controls (Fig. 4c, [mean (*EGFP* KD) = 1.00, mean (*Dr-SLC38A9* KD) = 0.284, t(14) = 4.40, *P* = 0.00061]). Moreover, histological examination revealed that the testes of *Dr-SLC38A9* knockdown worms were smaller (Fig. 4l) than those of control worms (Fig. 4k). These results suggest that *Dr-SLC38A9* is also involved in the development of the testes during sexual induction. After 6 weeks of treatment, the percentage of *Dr-SLC38A9-*knockdown worms that had developed a genital pore was significantly lower than that of controls (Table 1; Fig. 4g, h). The percentage of *Dr-SLC38A9-*knockdown worms that had developed a copulatory apparatus at this time point was higher than that after 4 weeks of treatment (Table 1; Fig. 4e, f). These results suggest that knockdown of *Dr-SLC38A9* delayed the development of the copulatory apparatus. Moreover, at the 6-week time point, *Dr-SLC38A9-*knockdown worms contained significantly lower levels of the yolk gland marker *Dryg* than controls (Fig. 4d, [mean (*EGFP* KD) = 1.00, mean (*Dr-SLC38A9* KD) = 0.0374, t(14) = 3.15, *P* = 0.0071]). In addition, histological examination revealed that *Dr-SLC38A9* knockdown inhibited the development of yolk glands (Fig. 4m, n). Most importantly, these results suggest that the knockdown of *Dr-SLC38A9* delayed the development of all reproductive organs during sexual induction.

**Table 1:** *Dr-SLC38A9* RNAi knockdown during sexual induction

Treatment period	Test food^a^	Number of worms developing a pair of ovaries (%)^b^	Number of worms developing copulatory apparatus (%)^b^	Number of worms developing a genital pore (%)^b^
Week 4	*EGFP* dsRNA	29/29 (100)	21/29 (72.4)	0/29 (0)
	*Dr-SLC38A9* dsRNA	22/29 (75.9)*^c^	5/29 (17.2)**^d^	0/29 (0)
Week 6	*EGFP* dsRNA	18/18 (100)	18/18 (100)	14/18 (77.8)
	*Dr-SLC38A9* dsRNA	19/19 (100)	15/19 (78.9) n.s.^e^	4/19 (21.1)***^f^

^a^Asexual worms were fed minced *B. brunnea* worms supplemented with *EGFP* (control), daily or *Dr-SLC38A9* dsRNA for up to 6 weeks (summarized in Fig. 4)

^b^Development of ovaries, copulatory apparatus, and a genital pore were observed externally using a stereoscopic microscope

^c^Statistical significance was calculated using one-sided Fisher’s exact test (*P = 0.0052)

^d^Statistical significance was calculated using the Chi-squared test (**х^2^ (1, *N* = 58) = 17.8, *P* < 0.001)

^e^Statistical significance was calculated using Fisher’s exact test (*P* = 0.059, n.s.: not significant)

^f^Statistical significance was calculated using the Chi-squared test with Yates’ continuity correction (***х^2^ (1, *N* = 37) = 9.7, *P* = 0.0018)

### D-Tryptophan activates *Dr-SLC38A9* expression in the reproductive organs of sexual worms

**Fig. 5:**
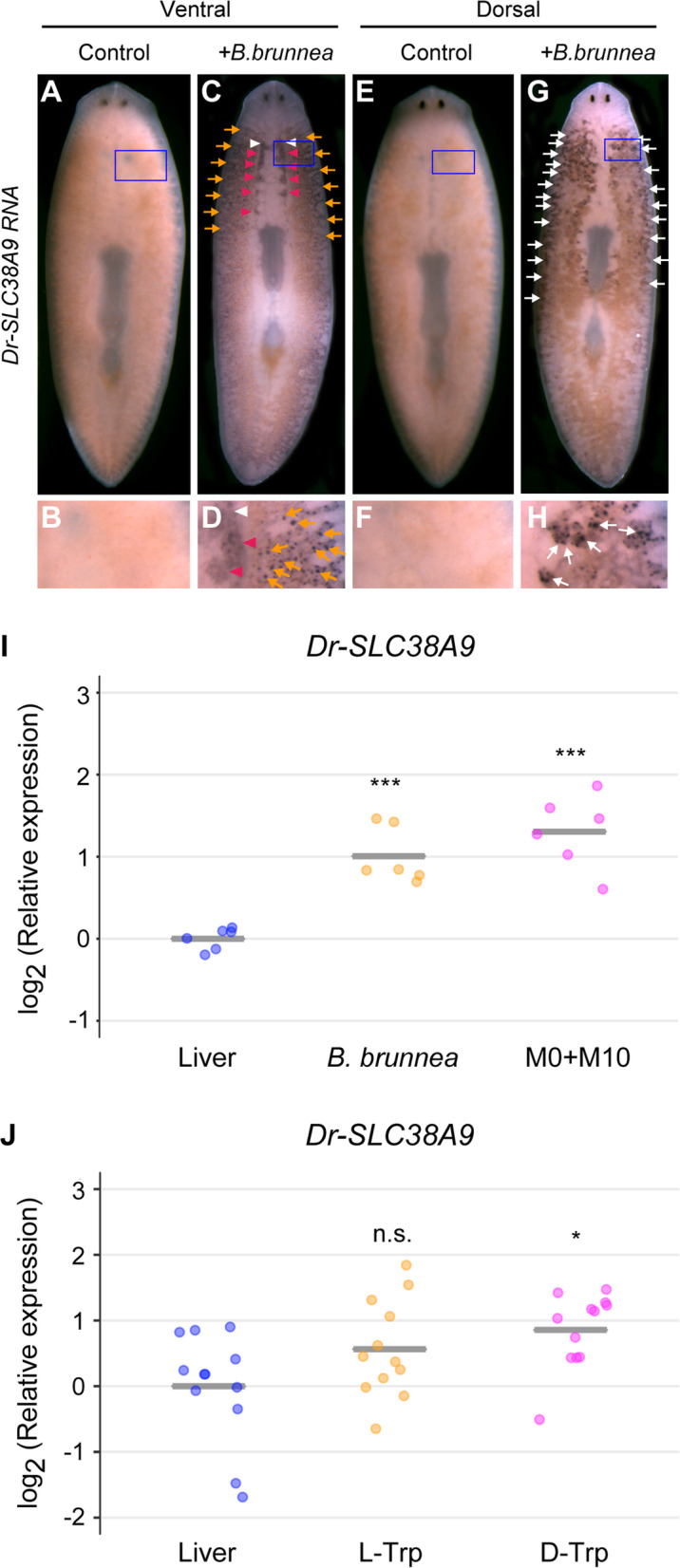
Induction of the expression of *Dr-SLC38A9* upon treatment with sex-inducing substances. **a**-**h** Whole-mount in situ hybridization of *Dr-SLC38A9* mRNA in sexual worms fed chicken liver (Control; **a**, **b**, **e**, **f**) or minced *B. brunnea* worms (**c**, **d**, **g**, **h**) daily for 3 days. *Dr-SLC38A9* transcripts are stained blue. Ventral views (**a**-**d**) and dorsal views (**e**-**h**) are shown, with the anterior part of the worm at the top. **b**, **d**, **f**, **h** Higher magnification views of areas inside blue squares in panels (**a**, **c**, **e**, and **g**). White arrowheads indicate signals in the ovaries (**c**, **d**). Magenta arrowheads indicate signals in the area where supernumerary ovaries will be formed (**c**, **d**). Orange arrows indicate signals in the yolk glands (**c**, **d**). Black arrows indicate signals in the testes (**g**, **h**). **i**, **j** qRT-PCR data are shown relative to the expression level (normalized to *Dr-ef1a* mRNA level) in the liver-fed worm, and log_2_ (relative expression) on the vertical axis indicates −∆∆Ct. The bars in the plots indicate the averages of −∆∆Ct. Asterisks indicate significant differences compared with liver-fed worms (Tukey’s HSD test: *P < 0.05; ***P < 0.001; n.s., not significant). **i** Six circles indicate individual samples of liver-fed worms, worms fed *B. brunnea,* and worms fed liver supplemented with the M0 + M10 fraction. **j** Twelve circles indicate individual samples of liver-fed worms and worms fed liver supplemented with l-Trp or d-Trp

Next, we investigated whether the expression of *Dr-SLC38A9* was related to the activity of sex-inducing substances. In this experiment, we chose sexual planarians as test worms because they contain many reproductive organs expressing *Dr-SLC38A9*. In the experimental sexual induction of asexual planarians, approximately 30 worms of the asexual planarians were fully sexualized by daily feeding with minced bodies of sexually mature *B. brunnea* worms (60 mg wet weight) for 4 weeks. We fed 15 sexual worms with approximately 120 mg wet weight of the sexually mature *B. brunnea* worms or chicken liver (control) over 3 days and carried out in situ hybridization analyses using a probe for *Dr-SLC38A9* RNA. Stronger *Dr-SLC38A9* expression was detected specifically in the ovaries (Fig. 5c, d; arrowheads), testes (Fig. 5g, h; white arrows), and yolk glands (Fig. 5c, d, g, h; orange arrows) of *B. brunnea*-fed sexual worms than those of control worms. In particular, in the ovaries, *Dr-SLC38A9* RNA was also detected in the area where supernumerary ovaries will be formed (Fig. 5c, d; magenta arrowheads) [19]. In this experiment, detection was halted according to the signal in *B. brunnea*-fed sexual worms, and due to the short detection time, no expression was detected in liver-fed sexual worms (Fig. 5a, b, e, f). Increased *Dr-SLC38A9* expression in *B. brunnea* was also confirmed by qRT-PCR (Fig. 5i, 2.01-fold). The question arose as to which chemical substance promoted *Dr-SLC38A9* expression. Previously, we established a procedure to obtain fractions containing sex-inducing substances (namely, the M0 + M10 fraction) from *B. brunnea* [19]. The M0 + M10 fraction derived from approximately 4 g wet weight of *B. brunnea* can induce full sexual induction for approximately 30 asexual planarians for a 4-week bioassay. Next, we investigated whether the expression of *Dr-SLC38A9* was related to the sex-inducing substances in the M0 + M10 fraction using qRT-PCR analysis. Fifteen sexual worms were fed daily with a piece of food containing M0 + M10 derived from approximately 1 g (wet weight) of *B. brunnea*, which contains a sufficient amount of sex-inducing substances, over 3 days. One-way between-subjects ANOVA was conducted to determine the effect of the sexual condition on *Dr-SLC38A9* expression. There was a significant effect of sexual condition on gene expression, with *P* < 0.001 for the three conditions [F(2, 15) = 25.16, *P* = 1.61e-05]. The resulting expression of *Dr-SLC38A9* was significantly enhanced by the M0 + M10 fraction of *B. brunnea* (Fig. 5i, 2.47-fold). This result suggests that the expression of *Dr-SLC38A9* is related to the activity of sex-inducing substances. Next, the question arose as to which chemical substance in the M0 + M10 fraction promoted *Dr-SLC38A9* expression.

Recently, we identified dl-tryptophan (Trp) as one of the sex-inducing substances contained in the M0 + M10 fraction [23]. We estimated that the M0 + M10 fraction from approximately 4 g (wet weight) of *B. brunnea* contains approximately 500 μg of l-Trp and approximately 2.5 μg of d-Trp, which was able to induce a pair of immature ovaries in the asexual planarians in the 4-week feeding-assay [23]. We investigated whether the expression of *Dr-SLC38A9* was related to the presence of l-Trp and d-Trp. Because the M0 + M10 fraction derived from approximately 1 g (wet weight) of *B. brunnea* significantly enhanced the expression of *Dr-SLC38A9* in 15 sexual worms in the 3-day bioassay (Fig. 5i), in this experiment, we set the dl-Trp content to be equal to the amount estimated to be present in the M0 + M10 fraction derived from approximately 1 g (wet weight) of *B. brunnea*. One-way between-subjects ANOVA was conducted to compare the effects of sexual condition on *Dr-SLC38A9* expression. There was a significant effect of sexual condition on gene expression, with *P* < 0.01 for the three conditions [F(3, 44) = 6.377, *P* = 0.0011]. As a result, worms fed with or without l-Trp did not show any statistically significant difference in the expression of *Dr-SLC38A9,* although the mean value was higher than that of the control (Fig. 5j, 1.48-fold). In contrast, when we fed sexual worms with d-Trp, the expression of *Dr-SLC38A9* was significantly higher than that in worms without d-Trp (Fig. 5j, 1.81-fold).

## Discussion

### *Dr-SLC38A9* is involved in the development of reproductive organs during planarian sexual induction

The deduced Dr-SLC38A9 protein possesses domains that are broadly conserved among SLC38A9 homologs. In addition, phylogenetic tree analysis comparing the sequence of Dr-SLC38A9 to representative human SLC38 family members supported that Dr-SLC38A9 is more closely related to SLC38A9 than to other SLC38 family members. Therefore, we concluded that *Dr-SLC38A9* is a homolog gene of human *SLC38A9*. We found that *Dr-SLC38A9* is expressed in the ovarian primordia of asexual worms. The ovarian primordia contain a small number of oogonia cells [1]. In sexual worms, *Dr-SLC38A9* was expressed during the early stages of germline differentiation, specifically in oogonia and spermatogonia, where the germline marker *Dr-nanos* was also expressed. Thus, *Dr-SLC38A9* is expressed in the early developmental stages of germline cells in both asexual and sexual worms. We demonstrated that knockdown of *Dr-SLC38A9* inhibited male and female germline development. Taken together, these data suggest that it is possible that the Dr-SLC38A9 protein activates the differentiation of oogonia and spermatogonia in gonads. We also found that *Dr-SLC38A9* was widely expressed in yolk glands and demonstrated that Dr-SLC38A9 knockdown inhibited yolk gland development. This suggests that the Dr-SLC38A9 protein activates the differentiation of yolk gland cells. Most importantly, the Dr-SLC38A9 protein is involved in the development of both the gonads and an important somatic reproductive organ, the yolk gland. In this study, knockdown of *Dr-SLC38A9* did not completely inhibit the development of these organs. This could be because the knockdown of *Dr-SLC38A9* resulted in only a 50% reduction in *Dr-SLC38A9* expression.

### *Dr-SLC38A9* is expressed in the dorsal midline at the prepharyngeal region in asexual worms

We found that *Dr-SLC38A9* is also expressed in the dorsal midline of the prepharyngeal region of asexual and sexual worms. This region is known to express a planarian *piwi* homolog, *Drpiwi-1*, which is a marker gene of germline cells as well as pluripotent stem cells [30]. In *Dugesia japonica*, there is a subpopulation of pluripotent stem cells in the same region defined by *DjPiwi-1,* another *piwi* family member that is not homologous to *Drpiwi-1* [39]. Therefore, we speculate that *Dr-SLC38A9-*expressing cells in the dorsal midline at the prepharyngeal region might be a subpopulation of pluripotent stem cells that have the potential to differentiate into germline cells. This speculation is supported by two pieces of evidence. First, when we fed asexual worms food supplemented with d-Trp for 7 weeks, a *Dr-nanos-*positive cell mass was induced at the dorsal midline (Fig. S3A, B, D), although during normal sexual induction, the expression of *Dr-nanos* was not found in the dorsal midline (Fig. S3E). Second, an ectopic *Dr-nanos* signal was detected in the same region of starved sexual worms, although this signal was not detected in normal sexual worms [40]. Starved sexual worms have no externally recognizable ovaries or copulatory apparatus, but they become fully sexual after being fed chicken liver once a week [40]. Thus, it is possible that *Dr-nanos-*positive cells in the dorsal midline at the prepharyngeal region contribute to sexual development. In future research, we will investigate whether *Dr-SLC38A9* is expressed in the dorsal midline at the prepharyngeal region of starved sexual worms.

### *Dr-SLC38A9* expression is enhanced by d-Trp

In the present study, we also demonstrated that *Dr-SLC38A9* expression is related to the activity of d-Trp. Interestingly, d-Trp was able to enhance the expression of *Dr-SLC38A9* as well as the M0 + M10 fraction. Therefore, the *Dr-SLC38A9* expression-inducing substance in the M0 + M10 fraction is considered to be d-Trp, although it is possible that other *Dr-SLC38A9* expression-inducing substances are also included. Thus, we propose that d-Trp activates *Dr-SLC38A9* function in reproductive organs. Moreover, we demonstrated that l-Trp did not promote SLC38A9 expression, because although the average expression in l-Trp-fed worms was higher than that of controls, the difference was not statistically significant. Both l-Trp and d-Trp have ovarian-inducing activity [23], although their effective pathways could be different. In fact, it has been suggested that l-Trp is metabolized to serotonin and functions in ovarian induction [27]. l-Trp can be converted to d-Trp by racemase activity; however, tryptophan racemase has not been isolated from animals. Considering the differences in the activity of l-Trp and d-Trp, if such a racemase even exists, its function is presumed to be weak.

### Molecular function of *SLC38A9*

Transporters play key roles in many biological processes that are regulated by amino acids [41]. The solute carrier (SLC) superfamily is the largest group of membrane transport proteins and comprises 55 gene families, having at least 362 putatively functional protein-coding genes in the human genome [42]. In humans, the SLC38 family consists of 11 members and encodes amino acid transporters known as sodium-coupled amino acid transporters (SNATs) [43]. Members of the SLC38 family are functionally classified as neutral amino acid transporters [43]. One of the genes in this family is involved in reproduction and embryogenesis, and mouse *Slc38a4* is important for maternal placentation [44]. As mentioned above, d-Trp can activate *Dr-SLC38A9* expression. In *Arabidopsis*, expression of the ATP-binding cassette transporter gene *AtABCG40* is enhanced by its substrate, abscisic acid [45]. This suggests the possibility that the substrate activates its transporter. Therefore, we speculate that d-Trp might be a substrate of Dr-SLC38A9. This speculation is supported by the fact that *Dr-SLC38A9* is expressed throughout yolk glands, which contain a large amount of Trp [23]. Recently, it was reported that human SLC38A9 transports l-arginine (l-Arg) and acts as an Arg sensor [36, 37]. SLC38A9 function is essential for the activation of mammalian target of rapamycin complex 1 (mTORC1) [36, 37], which controls cell growth and proliferation [46]. Therefore, we also speculate that Dr-SLC38A9 may act as a d-Trp sensor to control cell growth and proliferation during sexual induction. The current study suggests that d-Trp is taken directly into the ovaries and testis via the Dr-SLC38A9 transporter. Nevertheless, the biological function of d-Trp in the ovaries and testes remains unknown. A possibility is that it might act as a ligand for nuclear receptors. Additionally, qPCR analysis implied that *TR33723 | c0_g1_i1* is also highly expressed in the yolk glands, and *TR37685|c0_g2_i3* is expressed in the ovaries. We consider that in future works, it will be important to study whether *TR33723|c0_g1_i1*, *TR37685|c0_g2_i3*, and Dr-SLC38A9 proteins are capable of transporting d-Trp.

## Conclusions

d-Trp was the first substance to be identified as sex-inducing in the sexual induction of planarian *D. ryukyuensis*. In the present study, to investigate the mechanism of sexual induction of planaria by d-Trp, we isolated a putative amino transporter gene, *Dr-SLC38A9*, activated by d-Trp. Considering its expression and function, *Dr-SLC38A9* could play a role in the early developmental stages of germline cells and yolk glands. Its function in yolk glands could be related to the enrichment of Trp therein. Thus, the functional analysis of *Dr-SLC38A9* in this study is an important first step in clarifying the mechanism of planarian sexual induction by d-Trp.

## Supplementary Information

**Additional file 1: Figure S1.** Five stages of sexualization in the planarian *D. ryukyuensis*. The OH strain of the planarian *D. ryukyuensis* begins to develop reproductive organs upon switching from the asexual to the sexual state. The process can be roughly divided into five stages based on the development of reproductive organs [18]. Briefly, the asexual worm has ovarian primordia (op) at the ventral side. In stage 1, the ovary (ov), with an increasing number of oogonia, starts to form and becomes externally apparent. In stage 2, maturing ovaries with developing oocytes form. In stage 3, the primordial testis (te) at the dorsal side and primordial yolk glands (yg) at the ventral sides [35] form, and the copulatory apparatus (ca) becomes externally apparent at the ventral side. In stage 4, the genital pore (gp) becomes externally apparent at the ventral side. In stage 5, the testis (te) on the dorsal side and the yolk glands (yg) on both the ventral and dorsal sides mature. The worm is then ready for mating and egg laying. Note that the planarian body size changes because of the feeding procedure used for sexual induction [1]. If the food does not contain sex-inducing substances, asexual worms become larger without undergoing reproductive development.

**Additional file 2: Figure S2.** Nucleotide sequence of the *Dr-SLC38A9* gene and its predicted amino acid sequence. The isolated cDNA has a 78-bp 5′ untranslated region and an 84-bp 3′ untranslated region. A polyadenylation signal (AATAAA) is located 13 bp upstream of the beginning of the poly(A) tail. *Dr-SLC38A9* contains an open reading frame encoding a 585-amino acid polypeptide.

**Additional file 3: Figure S3.** Ectopic germline induction by d-Trp administration in the dorsal midline. (A, B) After we fed asexual worms food supplemented with d-Trp for 7 weeks, a serial sagittal section of a d-Trp-fed worm was prepared. (A) A section stained with hematoxylin and eosin. (B) In situ hybridization of *Dr-nanos* using an adjacent section. Red arrowheads represent a germ-like cell mass with a positive *Dr-nanos* signal. The dorsal side is at the top. In, intestine. Scale bar, 100 μm. (C–E) Whole-mount in situ hybridization of *Dr-nanos*. The test worms were fed (C) chicken liver homogenate (control) and (D) Trp for 7 weeks. (E) Stage 3 worms. Red arrowheads represent *Dr-nanos* expression in the dorsal midline at the prepharyngeal region, whereas blue arrowheads represent *Dr-nanos* expression in the presumptive region of the testes. Scale bars, 2 mm. Red and blue arrowheads represent *Dr-nanos* expression in the dorsal midline at the prepharyngeal region and presumptive region of the testes. These ectopic germlines were induced by administration of l-Trp, as well as d-Trp.

**Additional file 4: Supplementary Table S1.** Primer sets used for qRT-PCR.

**Additional file 5: Supplementary Data Sheet S1.** The raw data of qRT-PCR analyses for Fig. 1, 4 and 5.

## Data Availability

Data sharing is not applicable to this article, as no datasets were generated during the current study.
