# Supplementary figures and images for: d-Tryptophan enhances the reproductive organ-specific expression of the amino acid transporter homolog Dr-SLC38A9 involved in the sexual induction of planarian Dugesia ryukyuensis
Source: Zoological Lett. 2021 Mar 20;7:4. doi: 10.1186/s40851-021-00173-z (PMC7981857; doi:10.1186/s40851-021-00173-z)

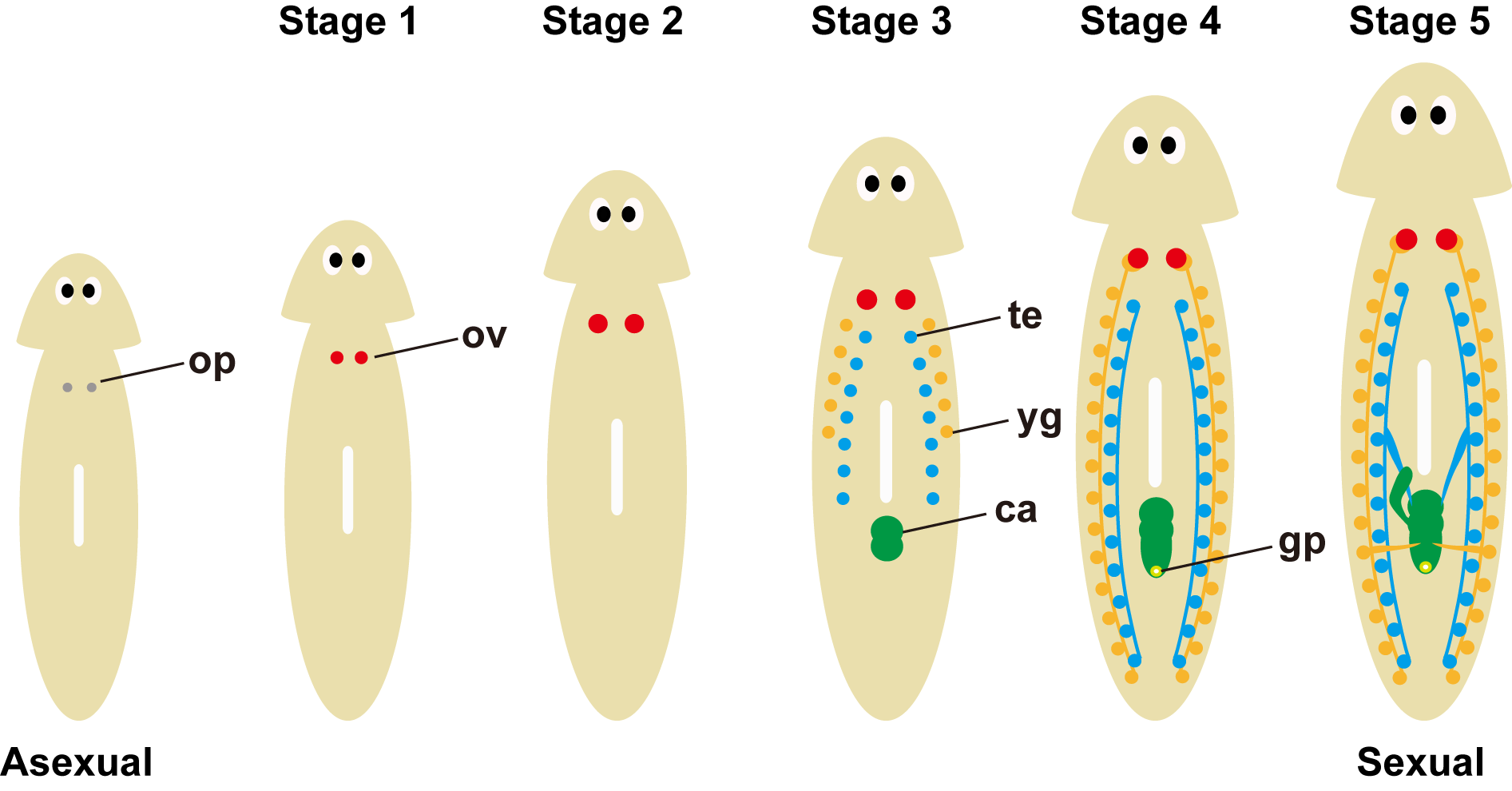

Supplement: Supplementary file 1 — Additional file 1: Figure S1. Five stages of sexualization in the planarian D. ryukyuensis. The OH strain of the planarian D. ryukyuensis begins to develop reproductive organs upon switching from the asexual to the sexual state. The process can be roughly divided into five stages based on the development of reproductive organs [18]. Briefly, the asexual worm has ovarian primordia (op) at the ventral side. In stage 1, the ovary (ov), with an increasing number of oogonia, starts to form and becomes externally apparent. In stage 2, maturing ovaries with developing oocytes form. In stage 3, the primordial testis (te) at the dorsal side and primordial yolk glands (yg) at the ventral sides [35] form, and the copulatory apparatus (ca) becomes externally apparent at the ventral side. In stage 4, the genital pore (gp) becomes externally apparent at the ventral side. In stage 5, the testis (te) on the dorsal side and the yolk glands (yg) on both the ventral and dorsal sides mature. The worm is then ready for mating and egg laying. Note that the planarian body size changes because of the feeding procedure used for sexual induction [1]. If the food does not contain sex-inducing substances, asexual worms become larger without undergoing reproductive development. [file 40851_2021_173_MOESM1_ESM.tif]

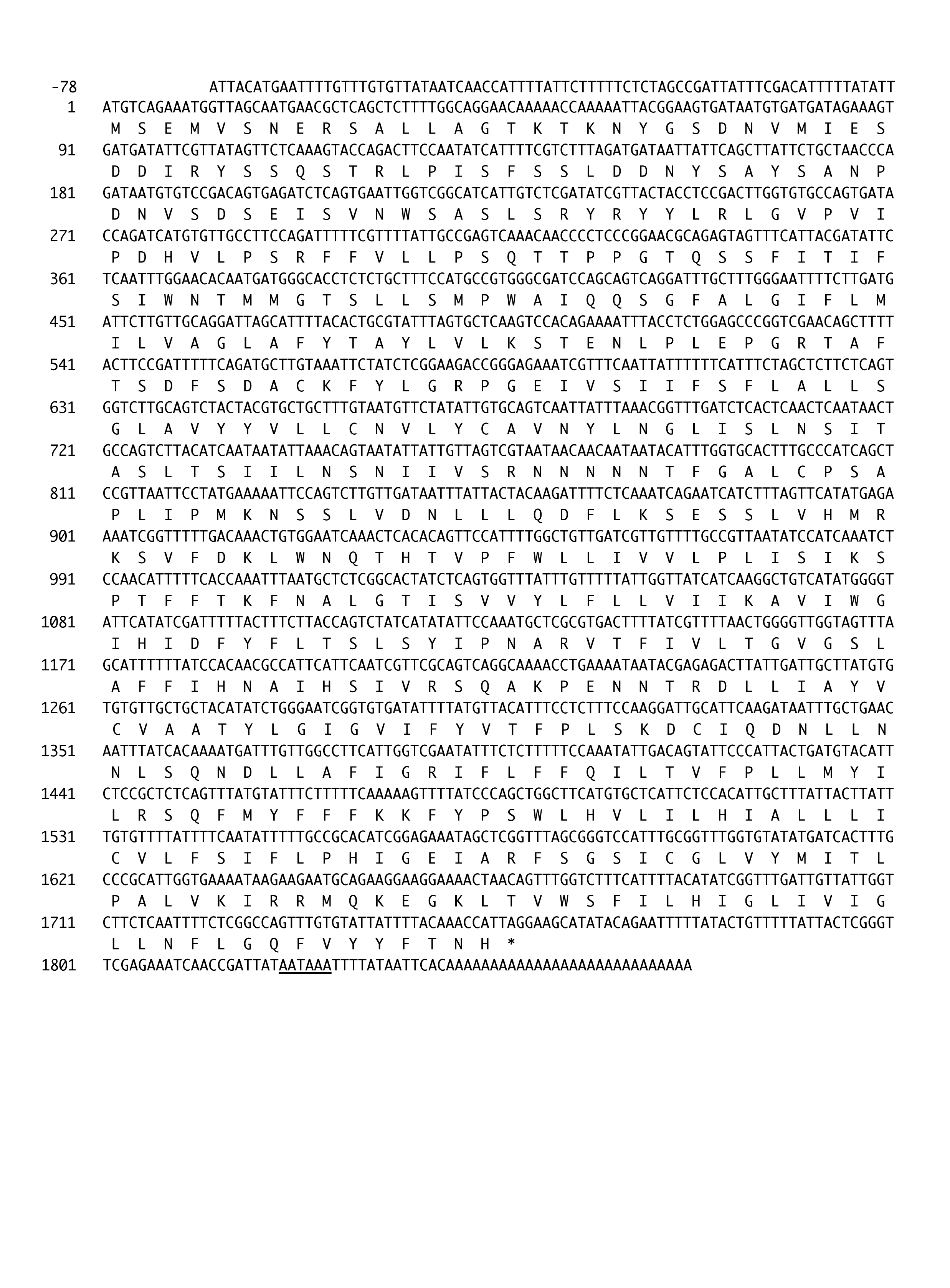

Supplement: Supplementary file 2 — Additional file 2: Figure S2. Nucleotide sequence of the Dr-SLC38A9 gene and its predicted amino acid sequence. The isolated cDNA has a 78-bp 5′ untranslated region and an 84-bp 3′ untranslated region. A polyadenylation signal (AATAAA) is located 13 bp upstream of the beginning of the poly(A) tail. Dr-SLC38A9 contains an open reading frame encoding a 585-amino acid polypeptide. [file 40851_2021_173_MOESM2_ESM.tif]

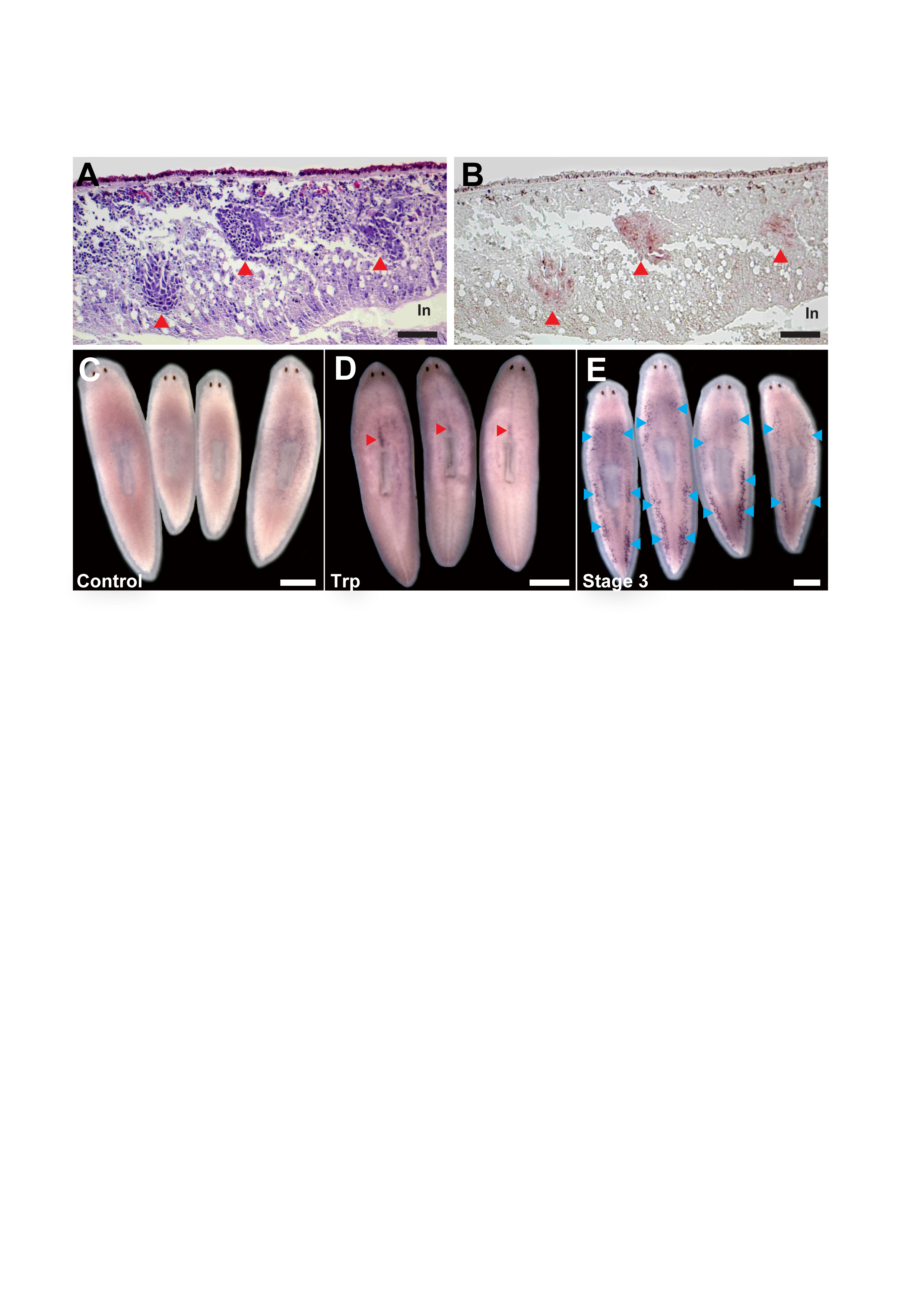

Supplement: Supplementary file 3 — Additional file 3: Figure S3. Ectopic germline induction by d-Trp administration in the dorsal midline. (A, B) After we fed asexual worms food supplemented with d-Trp for 7 weeks, a serial sagittal section of a d-Trp-fed worm was prepared. (A) A section stained with hematoxylin and eosin. (B) In situ hybridization of Dr-nanos using an adjacent section. Red arrowheads represent a germ-like cell mass with a positive Dr-nanos signal. The dorsal side is at the top. In, intestine. Scale bar, 100 μm. (C–E) Whole-mount in situ hybridization of Dr-nanos. The test worms were fed (C) chicken liver homogenate (control) and (D) Trp for 7 weeks. (E) Stage 3 worms. Red arrowheads represent Dr-nanos expression in the dorsal midline at the prepharyngeal region, whereas blue arrowheads represent Dr-nanos expression in the presumptive region of the testes. Scale bars, 2 mm. Red and blue arrowheads represent Dr-nanos expression in the dorsal midline at the prepharyngeal region and presumptive region of the testes. These ectopic germlines were induced by administration of l-Trp, as well as d-Trp. [file 40851_2021_173_MOESM3_ESM.tif]
