# Supplementary material for: d-Tryptophan enhances the reproductive organ-specific expression of the amino acid transporter homolog Dr-SLC38A9 involved in the sexual induction of planarian Dugesia ryukyuensis
Source: Zoological Lett. 2021 Mar 20;7:4. doi: 10.1186/s40851-021-00173-z (PMC7981857; doi:10.1186/s40851-021-00173-z)
Supplement: Supplementary file 4 — Additional file 4: Supplementary Table S1. Primer sets used for qRT-PCR. [file 40851_2021_173_MOESM4_ESM.docx]

**Supplementary Table S1. Primer sets used for qRT-PCR**

| Gene name | Forward primer (5'-3') | Reverse primer (5'-3') |
| --- | --- | --- |
| *GAPDH* | TTGATGTCGTTGCCTTGAAC | GACATTGCATGACCATCGAC |
| *Dr-ef1a/DrC_00434* | TTGGTTATCAACCCGATGGTG | TCCCATCCCTTGTACCATGAC |
| *TR33723* | CCCACGAATTTCCATTCAAG | GCTGGTAACAACGGATGAGC |
| *TR37685* | GCCTCAATTGGAGCATCAAG | TCTACGGGCAAATTGACAGATG |
| *TR38642* | CTGGCTTCATGTGCTCATTCTC | ATGCGGGCAAAGTGATCATA |
| *Dr-SLC38A9* | ATGGGCACCTCTCTGCTTTC | GGGCTCCAGAGGTAAATTTTCTG |
| *Dr-SLC38A9* | CTGGCTTCATGTGCTCATTCT | ATGCGGGCAAAGTGATCATA |
| *DrY1* (Testis marker gene) | TATGCCTCCACCTCCTCAAG | CGCCACGATAACCCATAATC |
| *Dryg* (Yolk gland marker gene) | AAATCTATCGTTGCCCGATG | TCGCATCGTTTTGATGTTTG |
